# Effect of polyelectrolyte complex formation on the antibacterial activity of copolymer of alkylated 4-vinylpyridine

**DOI:** 10.3906/kim-1909-95

**Published:** 2020-06-01

**Authors:** Murat TOPUZOĞULLARI

**Affiliations:** 1 Department of Bioengineering, Yıldız Technical University, İstanbul Turkey

**Keywords:** Polyelectrolyte complex, antibacterial, 4-vinylpyridine

## Abstract

Polymers bearing quaternized 4-vinylpyridine (QVP) groups are known for their antibacterial activities and these polymers can form polyelectrolyte complexes (PEC) with polyanions through electrostatic interactions. PEC formation can be used to adjust the antibacterial activity of polymers of QVP, deliver active molecules, or design antibacterial supramolecular structures. However, the antibacterial activity of PECs of QVP polymers has not been investigated. In this study, a copolymer of QVP was mixed with polyacrylic acid in various molar ratios of components to form PECs. Hydrodynamic diameters and zeta potentials of formed PECs were determined by dynamic and electrophoretic light scattering spectroscopy techniques. The zeta potentials of PECs changed between –24 and +16 mV with variation in the ratio of components. Antibacterial assays against *E. coli* revealed a relation of PEC formation with antibacterial activity since MIC values changed between 125–1000 μg/mL according to the ratio of components.

## 1. Introduction

Cationic polymers attract considerable interest in the field of biomedical applications as antibacterial agents due to their inherent bacteria-killing activity and easy synthesis and modification [1,2]. Polymers bearing quaternary ammonium groups are the most prominent polycations used for antibacterial purposes [3]. These polymers interact cooperatively with negatively charged groups of the bacterial membrane and kill bacteria by disrupting its membrane. Hydrophobic groups are also added to the polymer structure to increase the activity of polycations by interacting with hydrophobic alkyl chains of lipids [4]. However, hydrophobic groups in the structure of polycations may decrease the solubility of the polymer in aqueous solutions. Therefore, adding hydrophilic monomers or groups such as PEG, HEMA, or HPMA into the structure of the polycation becomes a crucial design parameter for higher antibacterial activity [5,6].

Copolymers of quaternized 4-vinylpyridine (QVP) with oligoethylene glycol methyl ether methacrylate (OEGMA) were investigated in depth by Youngblood and his colleagues with the purpose of developing biocompatible and effective antibacterial agents [5,7]. In the structure of QVP and OEGMA copolymer, while QVP and OEGMA monomers are the quaternized ammonium groups and hydrophilic groups, respectively, hexyl groups attached to QVP groups are the hydrophobic moieties. By changing the quantities of monomers and chain length of OEGMA, the polymer’s antibacterial activity and biocompatibility can be adjusted [8]. These polymers can also be used to develop more complex novel materials with the driving force of intermolecular interactions between the cationic structure of the polymer and an oppositely charged molecule such as pharmaceutical peptides/proteins, genetic materials, or natural/synthetic polyanions [9,10].

Electrostatic interactions between polycations and polyanions cause the formation of polyelectrolyte (or polyion) complexes (PEC). These complexes can be produced from synthetic polyelectrolytes and can also be found in nature, such as DNA-histone complexes. PECs have been investigated intensely for a long time in drug delivery applications [11], gene therapy studies [12], vaccine adjuvants [13], etc. Beyond these applications, PECs have been used as antibacterial materials, such as coatings [14] or hydrogels [15]. However, examples regarding the antibacterial activity of PECs are limited and a detailed correlation between the antibacterial activities of PECs with their properties needs to be studied in detail.

PECs are formed by cooperative electrostatic interactions between polycations and polyanions and the structure of the formed PECs depends on numerous parameters, such as the ratio of (+) and (–) charged groups, concentration, ionic strength of the solution, charge density of polyelectrolytes, chain length of polyelectrolytes, ionization degree of the charged groups, location of the charged group in the polymer structure, hydrophobic or hydrophilic groups in the polymer backbone, etc. [16]. In addition, PECs can reversibly dissociate to their polymeric components under certain conditions [17]. Thus, changes in the parameters of PEC formation or environmental conditions can affect size, shape, zeta potential, and consequently the biological activity of the PEC [18,19]. It is clear that the correlation of the composition and formation parameters of PEC with its antibacterial activity can induce the development of novel antibacterial materials with variable characteristics, which can also be sensitive to environmental stimuli.

In this study, an antibacterial cationic copolymer was used to form PEC with an anionic polymer and the effect of the complexation on antibacterial activity of the polycation was investigated. For this purpose, an antibacterial copolymer of QVP and OEGMA was synthesized and this copolymer was mixed with polyacrylic acid (PAA) to form PECs in varying mole ratios of polyelectrolytes. Physicochemical properties and antibacterial activity of the complexes were examined to reveal a relationship between the structure of the PEC and its biological activity.

## 2. Materials and methods

### 2.1. Materials

OEGMA_500_ (M_n_ = 500 g/mol), 4-vinylpyridine (4VP), and PAA (M_w_ = 30 kg/mol) were purchased from Aldrich. 4,4-azobis(4-cyanovaleric acid) (ACVA) and 1-bromohexane were obtained from Sigma-Aldrich. Diethyl ether and methanol were obtained from Merck. NaCl, NaH_2_PO_4_.5H_2_O, and Na_2_HPO_4_.12H_2_O were obtained from Riedel-de Haen. Ultra-pure water was obtained from a Millipore MilliQ Gradient system. All other chemicals used were analytical grade.

### 2.2. Equipment

^1^H-NMR spectra were acquired from Bruker Avance III 500 MHz and deuterated DMSO was used as the solvent.

GPC (Viscotek TDA302) with refractive index (RI) and right-angle light scattering (LS) detectors was used to determine the molecular weight and molecular weight distribution of poly(QVP-co-OEGMA). Eprogen CatSEC300 was the GPC column and the flow rate was 0.4 mL/min. A solution of 0.1 M acetic acid with 0.1 M NaCl was used as the mobile phase. Polyethylene oxide (M_n_ = 5 kg/mol, M_w_/M_n_ = 1.05) was used for the calibration of detectors. All samples were filtered by 0.45 μm syringe filters before GPC analysis.

The degree of quaternization of the copolymer was determined by FTIR spectrum acquired by Shimadzu IRPrestige-21 FTIR spectrometry with Pike MIRacle ATR device.

4VP groups of the copolymer were quaternized with microwave heating by using a Milestone Microsynth microwave oven. The reaction temperature of 80 °C was maintained by the preset maximum microwave energy of 200 W.

Hydrodynamic diameters (Dh) and zeta potentials of polymers and PECs were acquired by dynamic (DLS) and electrophoretic (ELS) light scattering spectrometry (Zetasizer Nano ZS, Malvern). All DLS and ELS measurements were acquired at 25 °C. General purpose (NNLS) analysis model was chosen from the data acquisition software of equipment for fitting the correlation function in DLS measurements. To determine their zeta potentials in ELS measurements, 1/10 diluted solutions of complexes were used.

### 2.3. Synthesis of poly(QVP-co-OEGMA) copolymer

A random copolymer of poly(4VP-co-OEGMA_500_) was obtained by polymerization of 4VP and OEGMA_500_ together. 4VP and OEGMA500 were dissolved in DMF with initial concentrations of 1.5 M and 0.17 M, respectively. The solution was purged with N_2_ gas for at least 30 min and the reaction vessel was tightly sealed. The mixture was heated to 70 °C and polymerization was initiated with the addition of ACVA. The concentration of ACVA was 5.6 mM. After 24 h, the polymer was precipitated with cold diethyl ether. The precipitate was dissolved and precipitated twice to remove the remaining monomer and DMF. The final product was dried overnight in vacuum at 50 °C and then characterized by GPC and ^1^H-NMR spectroscopy.

4VP groups of the copolymer were quaternized by the method used in our previous study [20]. Briefly, the obtained copolymer was dissolved in 2-propanol with a 10% concentration. 1-bromohexane was used as the quaternization agent and added to the solution with the mole number 5 times more than the 4VP groups in the dissolved copolymer. The reaction vessel, closed with a septum, was kept in the microwave oven for the reaction for 3 h. The reaction temperature was 80 °C, maintained by a maximum microwave energy of 200 W. The polymer was precipitated with cold diethyl ether and dried. The quaternized copolymer was characterized by FTIR spectrometry.

### 2.4. Preparation of complexes of poly(QVP-co-OEGMA) and PAA

Stock solutions of poly(QVP-co-OEGMA) and PAA were prepared separately in 0.02 M phosphate buffer at pH 7.0. Stock solutions were filtered with a 0.2 μm syringe filter. The stock solutions of polymers were mixed in various [NR^+^+_4_]/[COO^-^] ratios (0.1, 0.5, 1, 2, 3, 5, 10, 20, and 50). [NR^+^_4_]/[COO^-^] is the molar ratio of positively charged amine groups of poly(QVP-co-OEGMA) to negatively charged carboxylic acid groups of PAA. Eq. (1) shows the molar ratio calculation where C_poly(QV P−co−OEGMA)_ and CPAA are concentrations of polymers and M_poly(QV P−co−OEGMA)_ and M_PAA_ are molecular weights of polymers. In Eq. (1), while N_NR4+_ is the number of quaternized pyridine rings in one poly(QVP-co-OEGMA) chain, N_COO^-^_ is the number of ionized carboxylic acid groups in one PAA chain. pKa of PAA is 4.5 and all carboxylic acid groups in PAA chains are fully ionized at pH 7.0 [21]. In all PEC solutions, the concentration of poly(QVP-co-OEGMA) was 2 mg/mL. [NR^+^_4_]/[COO^-^] ratios were adjusted by changing the PAA concentration.

(1)[NR4+][COO-]=NNR4+.(Cpoly(QVP−co−OEGMA)/Mpoly(QVP−co−OEGMA))NCOO-.(CPAA/MPAA)

### 2.5. Antibacterial tests

The broth microdilution method [22] was used as the quantitative method to evaluate the antibacterial activity of polymers and PECs against *E. coli* (ATCC number: 25922). Polymer and PEC solutions were serially diluted in liquid growth medium using a 96-well plate. Minimum inhibitory concentration (MIC) and minimum bactericidal concentration (MBC) of samples were determined after incubation at 37 °C for 24 h. The bacterial culture medium (10^6^ cfu/mL) was added into each well and the final volume of the wells was adjusted to 200 μL. The negative control wells contained broth and bacteria only. While only broth was used for the blank control wells, a mixture of vancomycin antibiotic solutions (4 mg/L) and bacteria was used in positive control wells. MIC values were determined both spectrophotometrically (OD600 nm) and by a standard plate counting method.

## 3. Results and discussion

**Figure 1:**
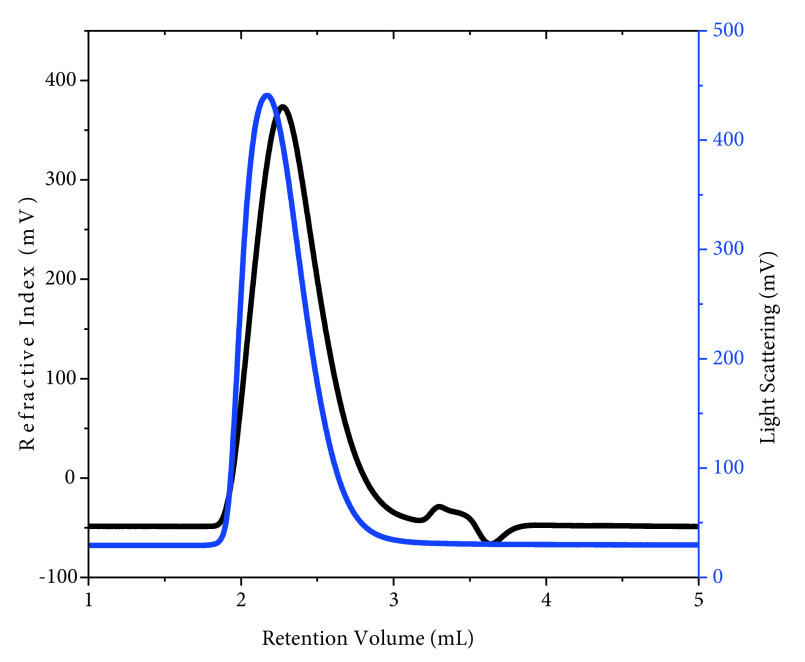
GPC chromatograms of poly(4VP-co-OEGMA) acquired from RI (black line) and LS (blue line) detectors.

The study investigates the effect of PEC formation on the antibacterial activity of poly(QVP-co-OEGMA). For this purpose, a random copolymer of 4VP and OEGMA500 was synthesized using free radical polymerization. The obtained copolymer was characterized by GPC and 1 H-NMR spectrometry. Figure 1 gives the GPC chromatograms of poly(QVP-co-OEGMA) acquired from RI and LS. As seen in Figure 1, the synthesized polymer has a unimodal distribution with PDI of 1.3 and Mw of the copolymer is 114 kg/mol.

**Figure 2:**
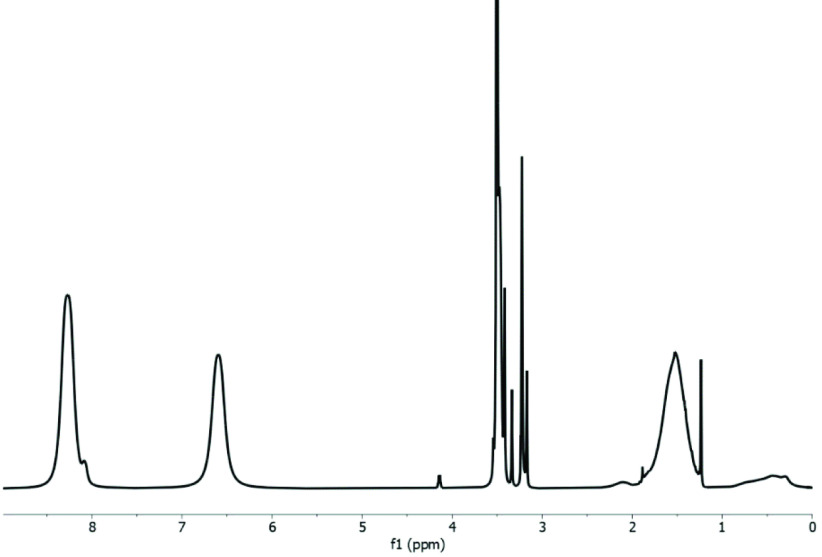
^1^H-NMR spectrum of poly(4VP-co-OEGMA) in DMSO.

In Figure 2, the ^1^H-NMR spectrum of the synthesized copolymer is given, in which protons of pyridine rings are clearly observed at 8.26 and 6.59 ppm. The peaks at 4.13 and 3.50 ppm belong to the CH_2_ protons on the side chain of OEGMA. Peak areas of these peaks allow determining the monomer quantities in the copolymer chain. Although 90 mol% of monomers were 4VP in the feed of polymerization, 97 mol% of the monomers in the final copolymer chain are 4VP and the remaining 3 mol% of monomers are OEGMA. This result complies with previous studies about the reactivities of these monomers [23,24], in which 4VP is more reactive than OEGMA and therefore less OEGMA monomers were included in the structure of the copolymer chain than the polymerization feed.

**Figure 3:**
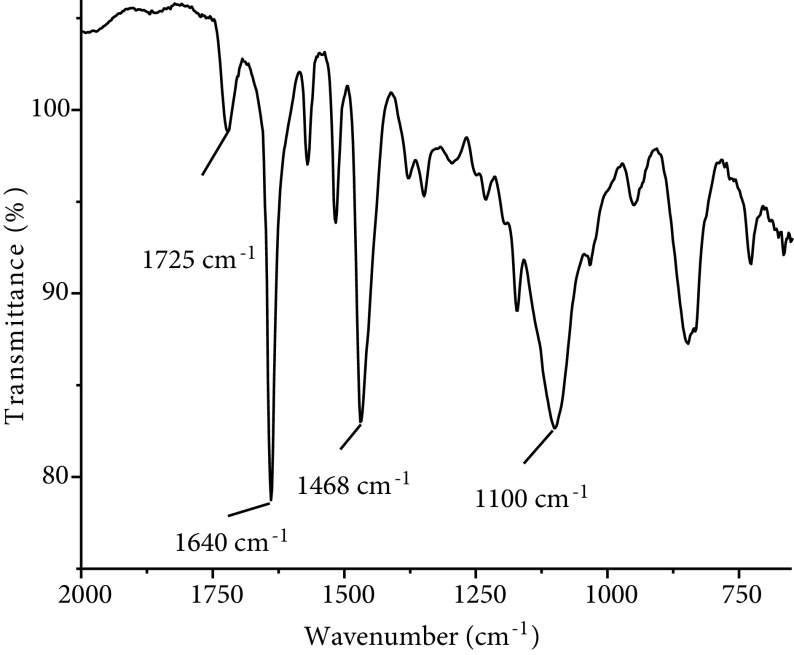
FTIR spectrum of quaternized poly(QVP-co-OEGMA).

To obtain a positively charged copolymer, amine groups of 4VP rings were quaternized with 1-bromohexane in the next step. Figure 3 gives the FTIR spectrum of the copolymer after the quaternization reaction. The FTIR spectrum allows to easily determine the quaternization and degree of quaternization of 4VP rings [20,25]. In Figure 3, the band at 1725 cm^-1^ belongs to the C=O group of the OEGMA monomer. Actually, C=O gives a strong and sharp band, but the molar ratio of OEGMA is very low in the copolymer chain according to the NMR spectrum. Therefore, a weak C=O band is observed in the copolymer’s FTIR spectrum. The band at 1100 cm^-1^ belongs to C–O–C ether groups in the side chain of OEGMA. Since there are 10 times more ether groups than C=O groups in the OEGMA monomer, the ether band gives a stronger band. The pyridine ring is characterized by the band at 1600 cm^-1^ , however, this band is observed as a very small shoulder next to the band at 1640 cm^-1^ , which belongs to the quaternized pyridine ring. As seen from the spectrum, the band of the quaternized pyridine ring at 1640 cm^-1^ gives a strong and sharp peak. The ratio of absorbances of the peaks at 1600 and 1640 cm^-1^ directly gives the degree of quaternization in the copolymer chain as 91% [20,25]. The band at 1468 cm^-1^ also shows the quaternized pyridine rings of the copolymer. The band of nonquaternized pyridine rings at 1415 cm^-1^ is not observed in the spectrum due to the high degree of quaternization.

The structure of poly(QVP-co-OEGMA) synthesized in this study depends on the polymer produced by Youngblood and his colleagues [5,7,8]. They showed that even 1% of hydrophilic monomers, such as OEGMA, in the structure of quaternized PVP dramatically increases the antibacterial activity of the polymer due to increased solubility and less interaction with proteins. Consequently, the polymer can interact with the membranes of bacteria. Alkyl groups attached to vinylpyridine rings are not only used for quaternization of the polymer, but also for increased interaction with hydrophobic lipid chains of the membrane to be disrupted. It has been shown that longer alkyl chains attached to the vinylpyridine group increase antibacterial activity [26]. However, solubility becomes a major problem when long alkyl chains are attached to the polymer after quaternization. Alkyl groups of up to 6 carbons used in quaternization of vinylpyridine are appropriate alkylating agents since their chain length is long enough to interact with lipids and short enough not to cause insolubility.

While antibacterial activity, biocompatibility, and effect of monomer composition or type of hydrophilic monomer on the antibacterial activity of the synthesized quaternized copolymer were studied extensively in previous studies [5,7,27], the effect of complexation with an oppositely charged molecule was not studied for this copolymer. Complexation with an oppositely charged molecule may allow a design of novel antibacterial materials delivering a different type of agents for more effective antibacterial applications or other drugs as dual effect systems. Also, the interaction of the copolymer with oppositely charged molecules under physiological conditions or using the copolymer for coating metallic or ceramic materials with electrostatic forces can affect the antibacterial activity of the copolymer. Therefore, in this study, we investigated the effect of the complexation of quaternized poly(4VP-co-OEGMA) with an oppositely charged polymer of PAA on the antibacterial activity of quaternized poly(4VP-co-OEGMA). For this purpose, we simply mixed the solutions of poly(QVP-co-OEGMA) with PAA in different molar ratios of [NR_+_^4^]/[COO^−^]. All solutions were clear, except for the slightly turbid solution of [NR_+_^4^]/[COO^−^] = 1.

Poly(QVP-co-OEGMA), PAA, and the complexes prepared in different molar ratios of these polymers were analyzed with dynamic and electrophoretic light scattering spectroscopy techniques to determine their hydrodynamic diameters and zeta potentials. In all samples, the concentration of poly(QVP-co-OEGMA) was fixed to 2 mg/mL and the concentration of PAA was changed to obtain the desired [NR_+_^4^]/[COO^−^] ratio.

**Figure 4:**
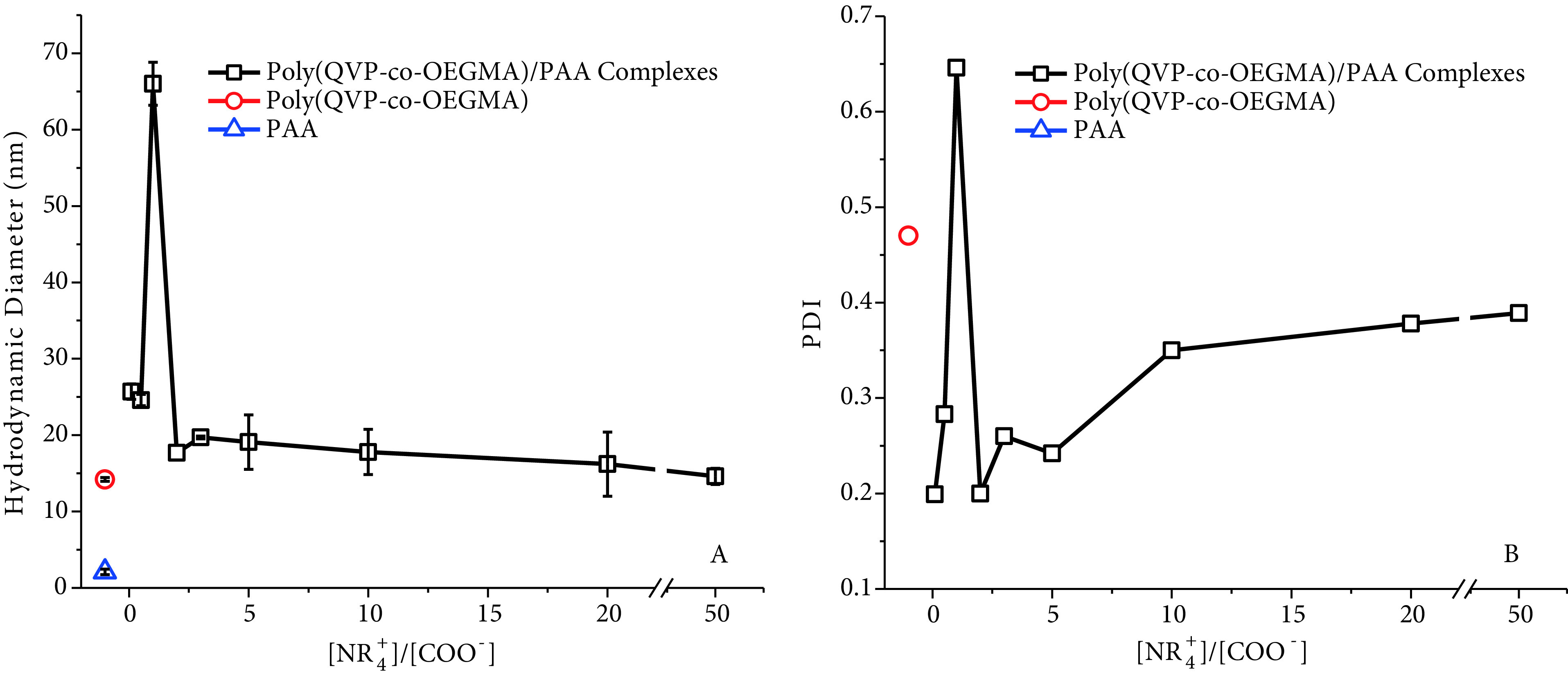
Hydrodynamic diameters (by volume) (A) and PDI values (B) of poly(QVP-co-OEGMA), PAA, and complexes of these polymers prepared in varying [NR_+_^4^]/[COO^-^] ratios.

Size and zeta potential are important physicochemical parameters that directly affect the biological activity of macromolecular systems. Figure 4 gives the hydrodynamic diameters and polydispersity index (PDI) values of the polymers and their complexes. While the diameters of poly(QVP-co-OEGMA) and PAA are 14.2 and 2.1 nm (Figure 4A), respectively, the PDI values of polymers are 0.47 and 0.34 for poly(QVP-co-OEGMA) and PAA (Figure 4B), respectively. These 2 polymers were mixed in varying [NR_+_^4^]/[COO^−^] ratios. In [NR+4]/[COO−] ratios of 0.1 and 0.5, hydrodynamic diameters close to 25 nm are larger than both poly(QVPco-OEGMA) and PAA, which reveals PEC formation with the help of intermolecular forces between oppositely charged polymers. In addition, PDI values of complexes prepared at [NR_+_^4^]/[COO^−^] = 0.1 and 0.5 ratios are lower than 0.3, which reveals a monodisperse particle formation [28] by mixing polydisperse polyelectrolytes. Poly(QVP-co-OEGMA) and PAA form PEC particles by binding together with electrostatic attractions.

Increasing the [NR_+_^4^]/[COO^−^] ratio to 1 caused the formation of larger PEC particles and turbidity in the solution, which can be a result of the neutralization of most of the charged groups with an oppositely charged group. This can lead to the formation of more hydrophobic structures and aggregation of PEC complexes together to form larger particles. In Figure 4A, while the diameter of the complex is given as 66 nm, there are also particles with a diameter of 516 nm. Relative abundances of these particles are 50.1% (66 nm) and 45.6% (516 nm), and the remaining particles are much larger aggregates (>5 μm). The PDI value of the complex at [NR_+_^4^]/[COO^−^] = 1 ratio is above 0.6, which exhibits the formation of random heterogeneous aggregates. The turbidity in the solution, large diameters of particles, and high PDI value observed at the ratio of [NR_+_^4^]/[COO^−^] = 1 reveal that a complex coacervation occurs at this ratio of polyelectrolytes. It is known that complex coacervation depends on the extended interactions between soluble complexes and coacervation tends to be maximized when the ratio of opposite charges in polyelectrolytes is close to 1 [29].

When the ratio was increased to [NR_+_^4^]/[COO^−^] ratios greater than 1, smaller PEC particles were formed with hydrodynamic diameters between 15–20 nm and PDI values between 0.2 and 0.39. All formed PEC particles, except the one with [NR_+_^4^]/[COO^−^] = 1, can have free charged chains not interacting with the oppositely charged groups and these free charged groups on PEC particles can repel other PEC particles and prevent the formation of larger aggregates. However, it is important to indicate that diameters and PDI values of PECs exhibit opposite trends above the ratio of [NR_+_^4^]/[COO^−^] = 1. Both the diameter and PDI values of complexes approach the values of free poly(QVP-co-OEGMA) as the ratio of components is increased above [NR_+_^4^]/[COO^−^] = 1. It can be said that the PEC exhibits characteristics of free poly(QVP-co-OEGMA) at very high [NR_+_^4^]/[COO^−^] ratios due to the fact that most parts of the polycation chain are free and not interacting with PAA.

**Figure 5:**
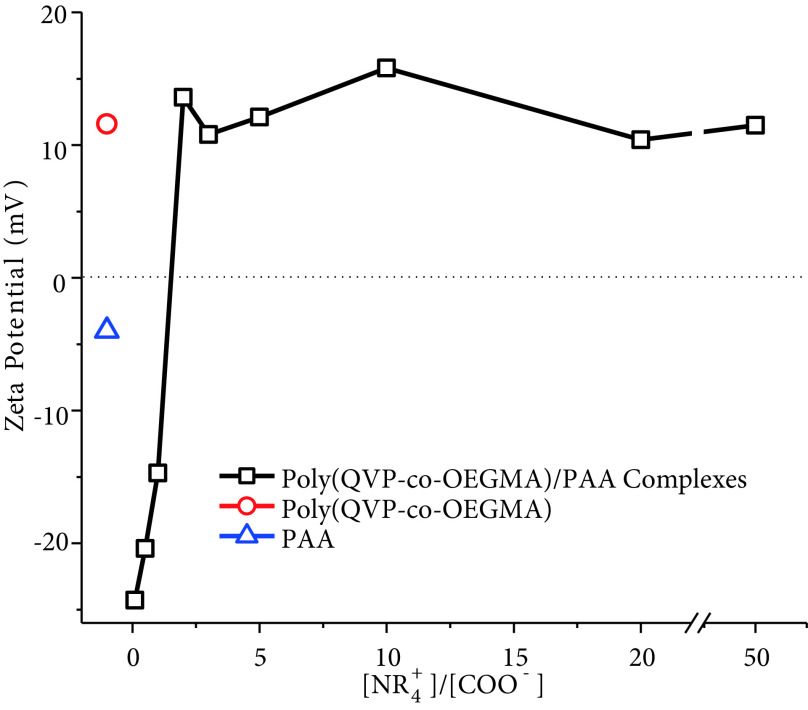
Zeta potentials of poly(QVP-co-OEGMA), PAA, and complexes of these polymers prepared in varying [NR_+_^4^]/[COO^-^] ratios.

Figure 5 shows the zeta potentials of individual polymers and the PECs formed by mixing oppositely charged polymers in different [NR_+_^4^]/[COO^−^] ratios. As expected, while the zeta potential of PAA is –4 mV, poly(QVP-co-OEGMA) has a zeta potential of +11 mV. The difference in absolute values (4 and 11) of the zeta potentials of PAA and poly(QVP-co-OEGMA) is the result of different molecular weights of the polymers and the number of charged groups on the related polymer chains. PECs with [NR_+_^4^]/[COO^−^] ratios lower than 1 have negative zeta potentials because of the free carboxylic acid groups in noninteracting PAA chains due to an insufficient number of positively charged QVP groups.

**Figure 6:**
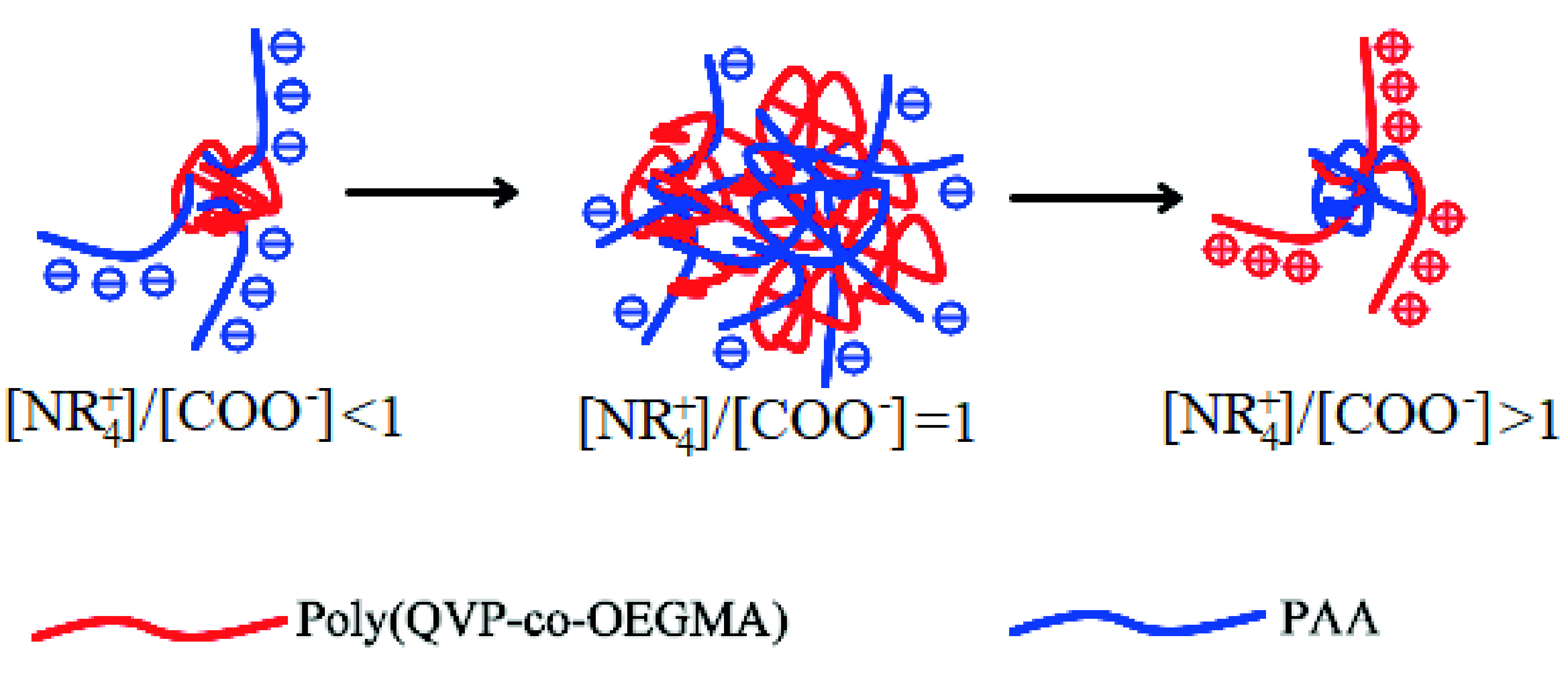
Schematic representation of formed PECs at different N/P ratios.

The PEC of [NR_+_^4^]/[COO^−^] = 1 has a zeta potential of –14.7 mV, which reveals that free PAA chains are available on the formed PEC particles. However, in this [NR_+_^4^]/[COO^−^] ratio, the numbers of oppositely charged groups are almost equal. Much bigger particles in solution refer to much more PAA and poly(QVP-co- OEGMA) that exist in a single PEC particle, and as a result, there can be a larger number of smaller free PAA fragments on these particles. In addition, these small PAA fragments on PEC particles do not have enough charge density to repel and form smaller PEC particles (Figure 6).

When the [NR_+_^4^]/[COO^−^] ratio was increased above 1, positively charged particles were obtained and the zeta potential value of PECs with [NR_+_^4^]/[COO^−^] ratios of 2 to 50 were between +10.5 and +15.8 mV. These values show the free poly(QVP-co-OEGMA) chains on the formed PEC particles that do not interact with PAA chains and repel other PEC particles to prevent the formation of larger aggregates.

Above and below [NR_+_^4^]/[COO^−^] = 1, oppositely charged groups of each polymer attract each other and form PEC particles. However, since the number of oppositely charged groups is not equal, there are noninteracting free charged groups in the PEC structure that repel other PEC particles.

At [NR_+_^4^]/[COO^−^] = 1, since there are almost equal numbers of oppositely charged groups, there is a stronger attraction between PAA and poly(QVP-co-OEGMA). In this ratio, formed PEC particles will be less charged and this may cause the particles to coalesce to form larger PEC particles, as observed in the size distribution. When less charged particles merge together, the charge of the final particle will be related to the sum of all these small numbers of charges. Here, it is significant to mention that environmental conditions, especially the ionic strength and pH, can directly affect the final PEC structure and zeta potential.

By changing the concentration of PAA and keeping the concentration of poly(QVP-co-OEGMA) constant, a set of PEC particles were obtained in a close range of hydrodynamic diameters (except [NR_+_^4^]/[COO^−^] = 1) but with different charges. This allowed us to observe the effect of PEC particle’s charge, size, and quantity of PAA on the antibacterial activity of poly(QVP-co-OEGMA). Table 1 gives MIC (minimum inhibitory concentration) and MBC (minimum bactericidal concentration) values of PAA, poly(QVP-co-OEGMA), and the PECs of these polymers against *E. coli* obtained from broth microdilution method. In Table 1, given MIC or MBC values are calculated from the concentration of poly(QVP-co-OEGMA).

**Table:** MIC and MBC values of PAA, poly(QVP-co-OEGMA), and PECs prepared with [NR_+_^4^]/[COO^-^] ratios from 0.1 to 50, acquired from the broth microdilution method.

Sample	MIC (μg/mL)	MBC (μg/mL)
Poly(QVP-co-OEGMA)	125	250
PAA	>1000	>1000
[NR_+_^4^]/[COO^−^]=0.1	500	1000
[NR_+_^4^]/[COO^−^]=0.5	1000	>1000
[NR_+_^4^]/[COO^−^]=1	>1000	>1000
[NR_+_^4^]/[COO^−^]=2	500	1000
[NR_+_^4^]/[COO^−^]=3	500	1000
[NR_+_^4^]/[COO^−^]=5	250	500
[NR_+_^4^]/[COO^−^]=10	125	250
[NR_+_^4^]/[COO^−^]=20	250	500
[NR_+_^4^]/[COO^−^]=50	250	500

In broth microdilution assays, PAA did not show any antibacterial activity against *E. coli* in the given concentration range. On the other hand, poly(QVP-co-OEGMA) had MIC and MBC values of 125 μg/mL and 250 μg/mL, respectively, which confirms the antibacterial activity of the copolymer against *E. coli* .

**Figure 7:**
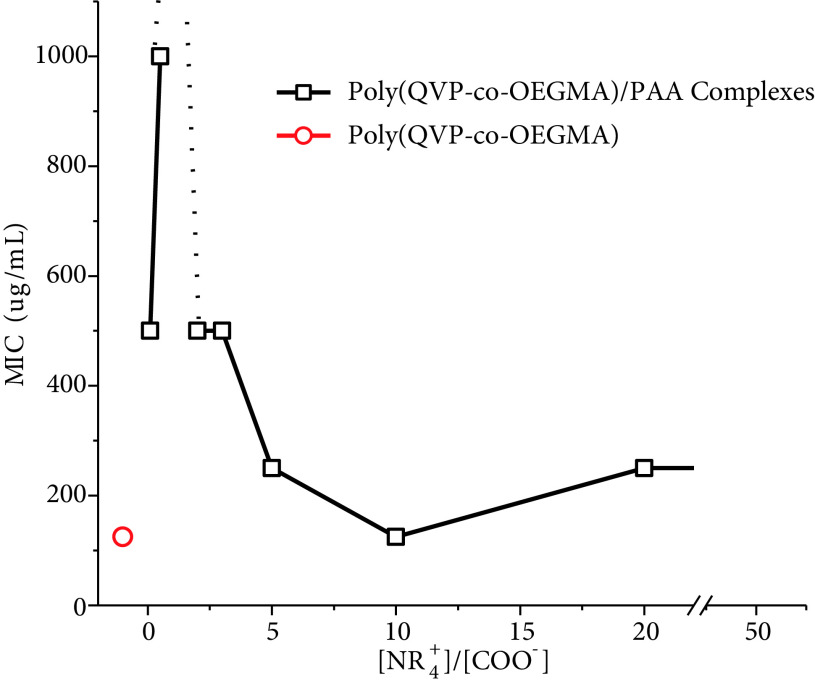
MIC values of poly(QVP-co-OEGMA) and PECs depending on the [NR_+_^4^]/[COO^-^] ratio.

When poly(QVP-co-OEGMA) was mixed with PAA in varying concentrations, the antibacterial activity of the copolymer changed with [NR_+_^4^]/[COO^−^] ratio (Figure 7). It is noteworthy that antibacterial activity was observed, although the PEC is negatively charged at [NR_+_^4^]/[COO^−^] ratios of 0.1 and 0.5. The mechanism behind this phenomenon can be explained by the fact that polyelectrolytes can migrate from one surface to another under certain conditions [30]. The dissociation of PECs by the migration of one of the components of PEC to another oppositely charged competitive polyelectrolyte was also studied in vivo by Mustafaev and coworkers [31]. In their studies, when a competitive polyanion was injected into mice just after the administration of an antigen containing PEC, PEC did not exhibit its function due to the dissociation of PEC by the migration of polycation of PEC to the competitive polyelectrolyte surface. Therefore, in broth medium, poly(QVP-co-OEGMA) can transit form PAA chains to a much larger (0.5–0.7 μm width and 1–3 μm length [32]) and negatively charged surface (–18.7 mV [33]) of *E. coli* and exhibit its antibacterial activity. The question arises when antibacterial activity of [NR_+_^4^]/[COO^−^] = 1 is not observed against *E. coli* . At [NR_+_^4^]/[COO^−^] = 1, more intermolecular attractions between PAA and poly(QVP-co-OEGMA) occur due to the almost stoichiometric complex formation. The higher number of intermolecular attractions causes stronger cooperative binding of polymers that prevented the transition of poly(QVP-co-OEGMA) to the bacterial cell membrane. At higher [NR_+_^4^]/[COO^−^] ratios from 2 to 50, formed PECs are positively charged and these positive charges are free quaternized 4VP groups that are not interacting with PAA due to insufficient number of acrylic acid groups. Therefore, the antibacterial activity of these complexes depends on these free groups and also on the migration of poly(QVP-co-OEGMA) to the bacterial surface. By increasing the [NR_+_^4^]/[COO^−^] ratio, the zeta potential of the complex becomes closer to free poly(QVP-co-OEGMA) and also the size of PEC becomes smaller, close to free poly(QVP-co-OEGMA) again. Therefore, PEC’s characteristics become similar to free polycation, which causes a similar antibacterial activity.

## Conclusion

PEC formation and the ratio of components of formed PECs affected the antibacterial activity of poly(QVPco-OEGMA) against *E. coli*. It is significant that both negatively and positively charged PECs exhibited MIC values, which can be related to the transition of the cationic copolymer from PAA to the cell membrane of bacteria. Only the [NR_+_^4^]/[COO^-^] ratio of 1 inhibited the antibacterial activity of the copolymer, which can be due to an increased number of intermolecular electrostatic interactions between poly(QVP-co-OEGMA) and PAA, preventing the interaction between poly(QVP-co-OEGMA) and the cell membrane of *E. coli*.

It has been shown in previous studies that copolymers of QVP and OEGMA can be both antibacterial and biocompatible, which allows these polymers to be used in vivo. Therefore, PEC complexes of QVP-OEGMA copolymers can be used to designate supramolecular structures with augmented antibacterial activity by delivering active pharmaceuticals or to adjust the biological activity of copolymer for balancing the antibacterial activity and biocompatibility. Also, PECs formed between QVP-OEGMA copolymers and genetic materials, such as antisense oligonucleotides, can exhibit dual effects in cancer patients by treatment of cancer and prevention of infectious diseases.
